# From Individual Behavior to Systemic Insight: A Bibliometric and Content Analysis of COM-B Applications in Responsible Consumption

**DOI:** 10.3390/bs16030474

**Published:** 2026-03-22

**Authors:** Olena Korohodova, Ionela-Andreea Puiu, Elena Druică

**Affiliations:** 1 The Research Institute of the University of Bucharest (ICUB), University of Bucharest, 030018 Bucharest, Romania; olena.korohodova@icub.unibuc.ro; 2 Department of Applied Economics and Quantitative Analysis, University of Bucharest, 030018 Bucharest, Romania; elena.druica@faa.unibuc.ro

**Keywords:** responsible consumption, COM-B, SDG 12, environmental concern, practical interventions

## Abstract

Understanding the psychological underpinnings of environmental decision-making is crucial for addressing climate change. Responsible consumption and pro-environmental behaviors often involve complex trade-offs between individual and collective outcomes, as well as between immediate and long-term consequences. Drawing on the Behavior Change Wheel and its core COM-B model—a comprehensive behavioral framework integrating Capability, Opportunity, and Motivation—this study systematically examines how the COM-B model has been applied in research on responsible consumption and environmentally relevant behavior. Using a combined bibliometric and content-analytic review of peer-reviewed studies indexed in the Web of Science between 2018 and 2026, we explore the focus, the behavior targets, and the contextual factors in existing COM-B applications. The findings reveal a focus on individual-level awareness, such as dietary behavior and sustainable lifestyles, while meso- and macro-level applications addressing institutional and policy mechanisms remain limited. By identifying a structural misalignment between the COM-B framework and its empirical applications, we contribute to behavioral science by highlighting the need to integrate structural determinants with individual processes to better understand and address the psychological mechanisms underpinning responsible decisions using this theoretical breadth. In this context, we emphasize the importance of aligning behavioral research priorities with the objectives of the United Nations Sustainable Development Goal 12.

## 1. Introduction

The global community faces increasing environmental and social pressures, underscoring the urgency of the United Nations Sustainable Development Goal 12 (SDG 12) on responsible consumption and production (World Bank, 2023), which calls for decoupling economic prosperity from resource depletion to ensure sustainable well-being (Bengtsson et al., 2018; Castellano et al., 2024). Despite growing environmental awareness and technological advances, favorable sustainability attitudes frequently fail to translate into consistent behavioral change (Barone et al., 2024; Nguyen et al., 2019). The persistent intention–behavior gap (Reppmann et al., 2025) is reflected in ongoing resource depletion, food waste, and unsustainable purchasing patterns. Although behavioral intention is widely recognized as a key predictor of pro-environmental behavior, its efficacy is often constrained by structural barriers, contextual limitations, and psychological dissonance (Duong, 2024; Saari et al., 2021). These patterns suggest that technological, regulatory, and economic instruments alone are insufficient as sustainable transitions. Ultimately, meaningful progress depends on a deeper understanding of the contextual and psychological mechanisms that shape how individuals, households, organizations, and communities make decisions and can be motivated to adopt responsible consumption practices.

In the context of implementing UN SDG 12, academic research has gradually shifted from focusing on isolated pro-environmental actions to examining the internalized behavioral components embedded within broader socio-ethical frameworks. Earlier scholarship often adopted a predominantly ecological perspective, which sometimes led to treating “environmentally relevant behaviour” and “responsible consumption” as overlapping or even synonymous concepts (Han, 2021; Hosta & Zabkar, 2021; Sharma et al., 2025). This approach resulted in a relatively narrow understanding of consumer responsibility. By contrast, the more recent literature argues that environmentally relevant behavior represents only one dimension of a broader construct of responsible consumption (Ertz, 2016; Han, 2021; Sharma et al., 2025). While environmentally relevant behavior generally refers to actions aimed at reducing environmental impact—such as resource conservation or waste reduction—responsible consumption encompasses a wider socio-ethical orientation that integrates environmental, social, and governance considerations (Hosta & Zabkar, 2021).

Moreover, the psychological mechanisms underlying these concepts differ. Environmentally relevant behaviors may, in some cases, operate as habitual or routine practices. Responsible consumption, by contrast, involves a conscious, volitional process in which individuals or organizations actively translate ethical intentions into consistent practices, often overcoming cognitive and contextual barriers (Bengtsson et al., 2018; Sheeran & Webb, 2016).

Accordingly, in this study “responsible consumption” is defined as a multidimensional behavioral strategy grounded in conscious ethical intention and aligned with promoting sustainable lifestyles. “Environmentally relevant behavior” is treated as a narrower subset of behaviors primarily concerned with environmental impact. This distinction is made across dimensions of scope (micro–meso–macro), intentionality (individual versus collective), and systemic impact.

Behavioral science provides a theoretically grounded pathway for addressing this challenge. Among the available frameworks, the Behavior Change Wheel (BCW) and its core Capability–Opportunity–Motivation–Behavior (COM-B) model have emerged as prominent tools for diagnosing behavioral determinants and informing the design of theory-driven, targeted interventions (Michie et al., 2014).

The COM-B model and BCW provide diagnostic precision by systematically capturing the psychological, social, and physical determinants of behavior. Their multilevel applicability has enabled effective implementation across diverse domains, including public health (D’Lima et al., 2020), pharmacy (Nielsen & Olsen, 2021), integrative oncology (Kwong et al., 2024), higher education (Toro-Troconis et al., 2021), and organizational change. Although originally developed within the public health context (Bîlbîie et al., 2021; Faija et al., 2021; Ng et al., 2023), these frameworks readily extend to other organizational contexts by facilitating the assessment of physical and social opportunities, as well as collective capabilities and motivation (Atkins & Michie, 2015; Michie et al., 2014). This conceptual versatility makes the model particularly well suited for understanding and guiding organizational transformation processes.

Recent comparative reviews (Bull et al., 2019; J. L. Davis et al., 2022; MacPherson & Kapadia, 2023) further indicate that interventions grounded in the COM-B model and BCW outperform traditional change approaches. By systematically addressing capability, opportunity, and motivation, these interventions achieve higher levels of engagement, effectiveness, and long-term sustainability, particularly within complex organizational environments. Moreover, integrating the COM-B framework into participatory intervention design enhances alignment between targeted behaviors and institutional realities, including those of public governance systems (Kalantari et al., 2024; Murphy et al., 2023; Rozskazov et al., 2021; Whittal et al., 2021).

Despite its growing relevance, research applying the COM-B model to responsible consumption remains conceptually fragmented, methodologically diverse, and dispersed across disciplinary domains. The existing studies have examined behaviors such as sustainable diets (Arrazat et al., 2024; Bertoni Maluf et al., 2024; N. Davis et al., 2024; Graça et al., 2023; Van Etten et al., 2023; Vezovnik & Kamin, 2024), waste reduction (Manika et al., 2022), water conservation (Addo et al., 2018), and green purchasing (Sundaraja et al., 2023), but these findings are rarely integrated across contexts. Moreover, the empirical research seldom provides a systematic overview of how the COM-B framework is operationalized across populations, settings, and intervention types. This conceptual and methodological fragmentation limits the accumulation of theoretically coherent insights and constrains the development of interventions that can be effectively transferred across different responsible consumption domains.

To address these gaps, this study presents a comprehensive bibliometric and content analysis of COM-B applications in the context of responsible consumption, drawing on peer-reviewed publications indexed in the Web of Science (WoS) between 2018 and 2026. The bibliometric analysis combines performance metrics with science-mapping techniques to characterize the structure of the field, to identify influential contributions, and to highlight both the dominant and emerging themes relevant to behavioral decision-making.

Complementing this, a content analysis synthesizes how behavioral determinants are conceptualized across 56 studies, how the COM-B model is operationalized, and which methodological approaches and levels of analysis are employed. By doing so, this study provides an evidence-based assessment of the conceptual coherence, methodological patterns, and overall relevance of COM-B applications within sustainable consumption contexts.

The contribution of this study is fourfold. First, it consolidates a fragmented body of evidence into a coherent overview of the field. Second, it maps the evolution of the COM-B applications in responsible consumption through rigorous bibliometric analysis. Third, it advances conceptual understanding by integrating thematic insights and behavioral diagnostics from content analysis. Finally, it offers practical guidance for policymakers, sustainability strategists, and researchers on designing behavior-change interventions that are informed by the key determinants of responsible consumption. To the best of our knowledge, this is the first study to combine bibliometric mapping with a theory-driven content analysis to systematically examine how the COM-B framework has been applied across responsible consumption research and SDG 12 targets.

## 2. Materials and Methods

### 2.1. Data

The data for this study were sourced from Clarivate Analytics’ Web of Science (WoS), a widely recognized scientific database that provides structured and reliable coverage of peer-reviewed research across disciplines. WoS was selected for its standardized metadata, transparent indexing criteria, and established use in research assessment and performance evaluation (Falagas et al., 2008; Mongeon & Paul-Hus, 2016). These features enhance methodological robustness, ensure the reproducibility of search and analytical procedures, and facilitate meaningful comparisons with existing bibliometric studies that utilize similar data sources.

Data identification, screening procedures, and eligibility assessments were carried out independently by two researchers, and disagreements were resolved through discussion. A structured search was conducted to identify studies applying the COM-B model and the Behavior Change Wheel framework within the context of consumption, with an emphasis on contributions relevant to SDG 12: Responsible Consumption and Production. The following Boolean search query was applied to the WoS Core Collection: ((“COM-B model” OR “COM B” OR “Capability Opportunity Motivation Behaviour” OR “Capability Opportunity Motivation Behavior” OR “Behaviour Change Wheel” OR “Behavior Change Wheel” OR “behavior change model”) AND (“consumption”)). This search yielded 204 publications, published in English. The titles, abstracts, and keywords were examined to determine conceptual relevance to behavioral framework and consumption-related phenomena. Through this screening, 89 publications were identified as relevant to this study’s thematic scope.

Subsequently, 19 publications were excluded because they did not address responsible consumption or other domains relevant to the scope of the analysis. Five additional publications were classified as partially relevant. Although they drew on behavioral models, their contribution to understanding responsible consumption behaviors was indirect (i.e., applying the COM-B/BCW framework to clinical or medical contexts rather than the “consumption” domain defined in our search query, or lacking an empirical focus on SDG 12 targets). These articles were retained for contextual purposes but were excluded from the core bibliometric dataset. Finally, nine publications were excluded due to restricted access, which prevented a full-text evaluation and verification of their relevance.

**Figure 1:**
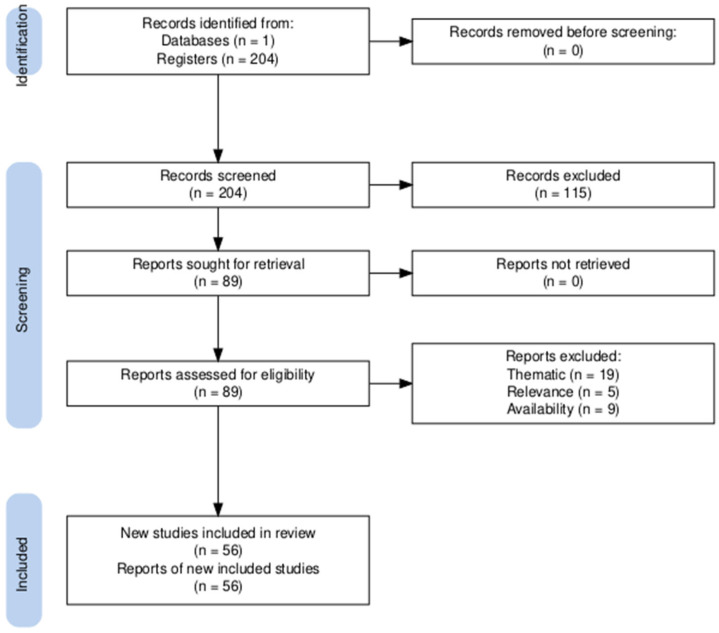
PRISMA Selection Protocol.

The final dataset comprises 56 publications that met all inclusion criteria and were deemed to be directly relevant to the scope of this analysis. The selection process is summarized in Figure 1.

### 2.2. Method

The research methodology integrates two complementary approaches: bibliometric analysis and content analysis. The concept of responsible consumption was operationalized through a combination of search strategy keywords and thematic indicators applied during the content analysis stage.

Bibliometric analysis is increasingly employed to examine research productivity and the intellectual structure of research in behavior change, sustainability, and responsible consumption research (Aria & Cuccurullo, 2017; Donthu et al., 2021). Within the field of responsible consumption, bibliometric methods have been applied to map the diffusion and application of behavior change frameworks, particularly the COM-B model, across domains including public health (Song, 2025), environmental studies (Zhu et al., 2021), and consumer research (Shekhar & Venugopal, 2025). Following established methodological approaches, the analysis combines performance analysis and science mapping (Cobo et al., 2011) to identify influential contributions, collaboration patterns, and emerging research themes related to COM-B-based interventions (Michie et al., 2014).

Our analysis was conducted using the Bibliometrix package (Aria & Cuccurullo, 2017) and its web interface Biblioshiny, within the R software environment (R Core Team, 2024), version 4.3.3. In addition, VOSviewer (Van Eck & Waltman, 2010), version 1.6.20, was employed to construct and visualize bibliometric networks.

Following the bibliometric mapping, a structured content analysis was conducted on the final sample of 56 articles to examine how the COM-B model is operationalized in responsible consumption research. Articles were coded using a predefined protocol combining automated keyword extraction with manual validation and binarization of thematic indicators (e.g., consumption, behavior change, green, diets). Each article was further classified by primary research design and by analytical level based on abstract and full-text screening. This procedure enabled a systematic comparison of dominant themes, target populations, research settings, and methodological patterns across the studies, while also identifying empirical and conceptual gaps in the application of the COM-B model to responsible consumption.

The research design combines a deductive logic of study selection with a partially inductive content analysis. The selection of studies was carried out based on inclusion criteria defined in advance, and based on existing theoretical approaches to responsible consumption and behavioral change. Only those publications that met the following criteria were included in the final sample: the use of the COM-B model or the Behavior Change Wheel and the presence of a clear link to consumption behaviors relevant to SDG 12. Elements of the inductive approach were applied in the subsequent stage of content analysis, where thematic categories were refined based on the empirical material.

## 3. Bibliometric Analysis

### 3.1. Performance Analysis

#### 3.1.1. Articles-Related Metrics

Bibliometric indicators, such as publication counts and citation measures, are widely used to assess scholarly productivity and influence within a research field (Aria & Cuccurullo, 2017; Passas, 2024). The final database comprises 56 publications (45 scientific articles, 9 reviews, and 2 early-access items) published between 2018 and 2026 across 37 distinct scientific sources (journals, books, etc.), resulting in a document-to-source ratio of approximately 1:5. Descriptive statistics are summarized in Table 1.

An average of 20.62 citations per document over a time span of only 2.16 years suggests a rapidly emerging research front (Cobo et al., 2011; Peña-Rocha et al., 2025; Zimmer, 2019). The observed annual growth rate (−12.83%) reflects the inclusion of the incomplete 2025 and early-access 2026 records, while trends prior to the cutoff suggest stable interest in the topic. The dataset comprises 269 unique authors, with an average of 5.27 co-authors per document, reflecting broad scholarly engagement and comparatively large research teams (Wuchty et al., 2007). International collaboration is further reflected by a co-authorship rate of 35.71%.

**Figure 2:**
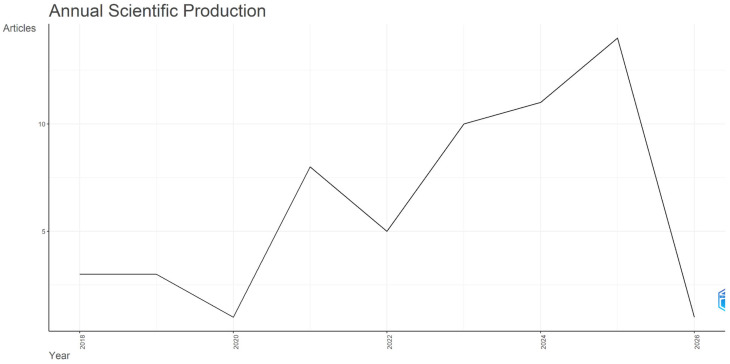
Time Span Scientific Production.

**Figure 3:**
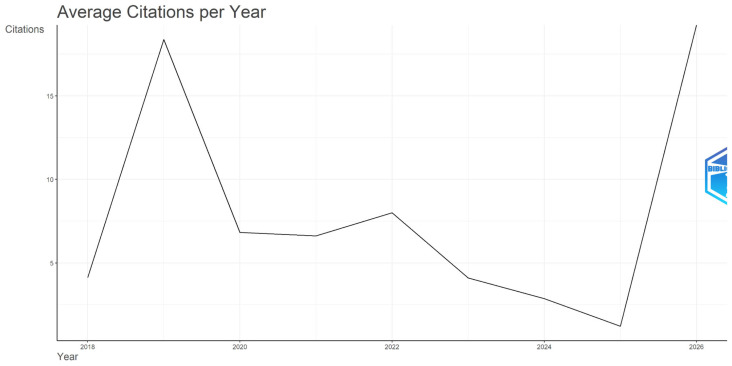
Longitudinal Overview of the Citation Metrics.

Annual scientific output from 2018 to 2026 exhibits an overall upward trend despite some fluctuations. Publication activity was low and stable in 2018 and 2019 with three documents each year, dropped to a single document in 2020, and then increased markedly from 2021 onward, with 8 publications in 2021, followed by 5 documents in 2022, 10 in 2023, and 11 in 2024. The field reached its peak productivity in 2025, with 14 publications. Research evolution over time is presented in Figure 2 and Figure 3.

Figure 3 illustrates that, despite only three publications in 2019, the average total citations per article reached 128.67, with an annual citation rate of 18.38 over seven citable years, suggesting the presence of highly influential contributors. Articles between 2018 and 2022 show relatively stable citation activity, with average total citation per article ranging from 32 to 41, and annual citation rates between 6.62 and 8.00. The citation metrics decline for articles from 2023 onward (12.3 in 2023, 5.73 in 2024, and 1.21 in 2025), reflecting an end-of-period truncation effect (Dass et al., 2017). In 2023, the average total citations per article fell to 12.3, and further declined to 5.73 in 2024 and 1.21 in 2025. This pattern is consistent with the time lag required for publications to accumulate citations, and represents a methodological expectation (Puiu & Bîlbîie, 2025) rather than an indication of declining research quality.

#### 3.1.2. Countries and Regions Scientific Production

The geographical and structural analysis of scientific production and collaboration, based on a full counting of country affiliations, reveals a network dominated by Anglophone countries. Consistent with prior evidence on global science networks (Wagner et al., 2015), the field exhibits a highly asymmetric structure, centered on the United States, the United Kingdom, and other Northern European countries. Within this landscape, the United Kingdom emerges as the primary hub (Department for Science, Innovation & Technology, 2025), combining the highest publication output (64 documents) with strong citation impact (256 citations; 17.1 citations per article), while serving as a connector across regions. The strongest bilateral collaboration is with Australia (37 publications; 13.6 citations per article), forming a trans-hemispheric axis of collaboration rooted in behavioral psychology and public health nutrition (Krahe et al., 2024). Additional over-represented collaborations include United Kingdom–Italy, the United Kingdom–Singapore, Italy–Vietnam, and Australia–New Zealand.

**Figure 4:**
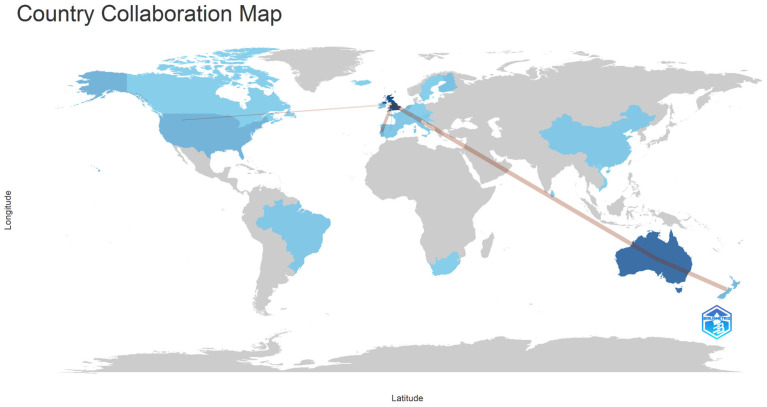
Country’s Collaboration World Map (full counting of author affiliations).

A secondary yet influential axis connects the United Kingdom with the United States (13 publications; 47 citations). Co-authorship mapping (Figure 4) underscores the centrality of the UK–Australia–USA triangle (Wondimagegn et al., 2023), alongside substantial European activity. Countries such as Portugal (22 publications; 443 citations; 110.8 citations per article) and the Netherlands (17 publications; 97 citations) contribute high-impact outputs, particularly in health system reform and behavioral intervention design. Sweden, despite a modest publication count (three), exhibits exceptional citation density (70 citations; 70 per article), highlighting the significance of targeted, high-visibility contributions.

The VOSviewer topological analysis (Appendix A) indicates a highly integrated field, with 24 of the 30 active countries (80%) forming a single interconnected component. Figure 4 illustrates the global collaboration network.

#### 3.1.3. Authors Performance

**Figure 5:**
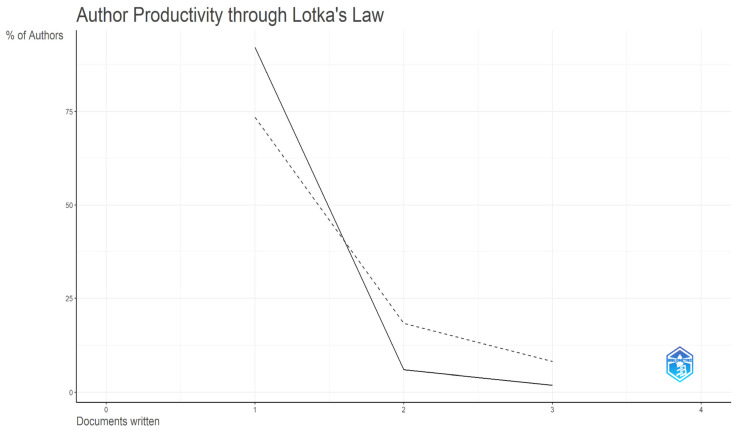
Author’s Productivity—Lotka’s Law.

The dataset comprises 269 distinct authors. To examine authorship structure, Lotka’s law of scientific productivity was applied to assess the distribution of authors by number of contributions. Figure 5 and Table A1 (Appendix B) show a highly skewed distribution.

According to the inverse square law, approximately 73.5% of authors are expected to have a single publication. However, the empirical data show a higher concentration of single-contribution authors (92.19%, n = 248). By contrast, only 1.86% of authors have produced three or more documents. Table A1 (Appendix B) reports the performance of the most prolific authors in the dataset.

#### 3.1.4. Institutions Performance

The raw dataset initially included 126 affiliation entries. Following a data cleaning process that excluded non-institutional records, such as individual author names and technical markers for corresponding authors, a total of 116 academic organizations were identified. Approximately 60% of these institutions contributed with only one article, as presented in Table 2.

Table 2 ranks the ten most productive academic institutions and reveals a marked geographical concentration of research within Europe and Australia/New Zealand. Although research output is distributed across multiple organizations, high-impact contributions are concentrated in established European hubs, such as Utrecht University (QS Rank 103) and the University of Helsinki (QS Rank 116), alongside specialized research centers in Australia (e.g., University of New England). The institutional landscape is dominated by Global North organizations, particularly those with strong expertise in behavioral psychology and environmental sustainability. The absence of leading US or Asian institutions suggests that the application of the COM-B model in consumption research is currently driven largely by a European research agenda, potentially shaped by EU sustainability initiatives, such as the Green New Deal (Allison et al., 2022; Craveiro et al., 2021; Fernandez-Alvarez et al., 2025; Ježovit & Lučić, 2025; Lee et al., 2025; Manika et al., 2022; Mezzacapo et al., 2025; Van Den Berg et al., 2022; Walker, 2024).

#### 3.1.5. Sources Analysis

Table 3 lists the top contributing journals by publication volume and citation impact, spanning behavioral nutrition (Appetite), food science (Food Quality and Preference, Trends in Food Science & Technology), and multidimensional sustainability (Sustainability, Journal of Cleaner Production). This distribution underscores the interdisciplinary nature of the research applying the COM-B model to responsible consumption.

**Figure 6:**
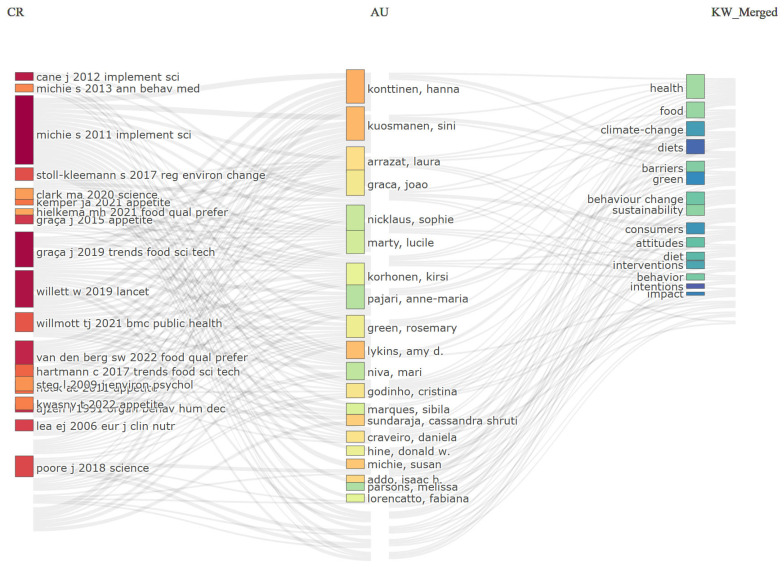
Sankey Diagram of Theoretical Foundations.

The local cited sources analysis highlights the prominent role of Implementation Science, which ranks third with 94 citations. Figure 6 illustrates how these theoretical foundations are reflected in the current research by linking frequently cited references (left), contributing authors (center), and dominant keywords (right). To enhance analytical objectivity and to minimize artefacts arising from the dominance of search terms, key query terms were excluded, allowing for a clear identification of the field’s underlying intellectual structure and latent thematic layers (Nowakowska, 2025; Zupic & Čater, 2015).

The left column highlights the foundational intellectual pillars of the field, dominated by Michie’s methodological frameworks on the Behavior Change Wheel (2011, 15 connections) and the classification of behavior change techniques (2013; 12 connections). These are closely integrated with influential work on planetary health and dietary sustainability by Willett et al. (2019, 11 connections) and Poore (2018, 9 connections). The middle column reveals a cohesive group of contributing researchers, led by Konttinen and Kuosmanen (15 connections), and Graça et al. (12 connections).

The flow towards the right column (keywords) highlights the dominance of terms such as “health” (18 connections), “food” (16 connections), and “sustainability” (14 connections). The prominent presence of “behaviour change” (11 connections), “interventions” (10 connections), and “barriers” (9 connections) indicates the application of implementation science to address the “intention-behaviour gap.”

**Figure 7:**
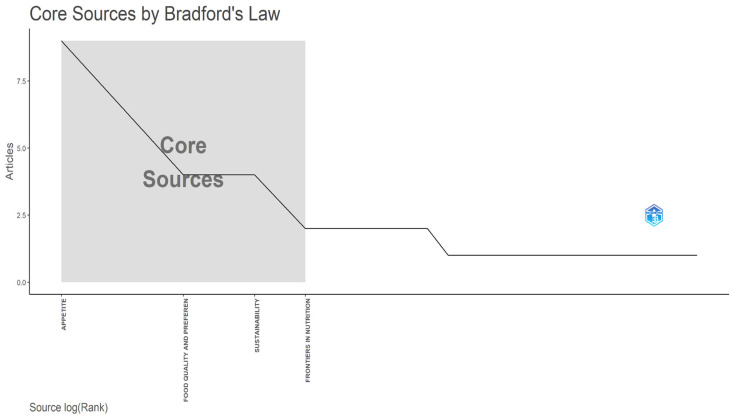
Core Sources—Bradford’s Law.

Bradford’s Law (Xue & Liu, 2024) reveals a significant concentration of the literature within a small “Core Zone” (Zone 1), as shown in Figure 7. This core comprises three journals (Appetite, Food Quality and Preference, and Sustainability), which together account for a significant share of the literature, anchoring the field at the intersection of consumer behavior, sensory science, and multidimensional sustainability. Frontiers in Nutrition leads the second zone, acting as a bridge between the core journals and the broader periphery of less frequently cited sources.

The cumulative growth of the most productive journals between 2018 and 2026, as shown in Table 4, reveals diverging publication trajectories from around 2020 onward. Appetite displays an exponential growth pattern, consolidating its status as the primary outlet in the field, with output accelerating markedly from 2021 after minimal activity in earlier years.

Following this trend, Food Quality and Preference and Sustainability show steady, linear growth, indicating sustained interest in the topic. Newer outlets, including Frontiers in Nutrition and the Journal of Cleaner Production, emerged after 2021–2022, reflecting both field maturation and expansion towards broader public health and environmental engineering perspectives. Overall, the upward trajectory across leading sources confirms that this research domain is in an active expansion phase.

#### 3.1.6. Keywords Analysis

The keyword analysis (Garfield, 1990; Zhang et al., 2016) shows that frequently used keywords and their root forms are closely aligned with the measurement of behavior change in responsible consumption using the COM-B model. Word clouds provide an intuitive overview of the textual data and serve as a useful exploratory tool for identifying dominant topics and initial thematic patterns, which can subsequently be examined using more advanced bibliometric techniques (Aria & Cuccurullo, 2017).

**Figure 8:**
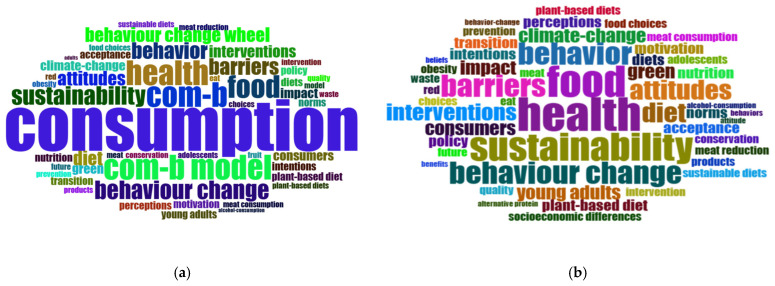
Word Cloud Analysis: (**a**) raw keyword frequency including search terms; (**b**) refined thematic focus after removing the primary search criteria.

**Figure A2:**
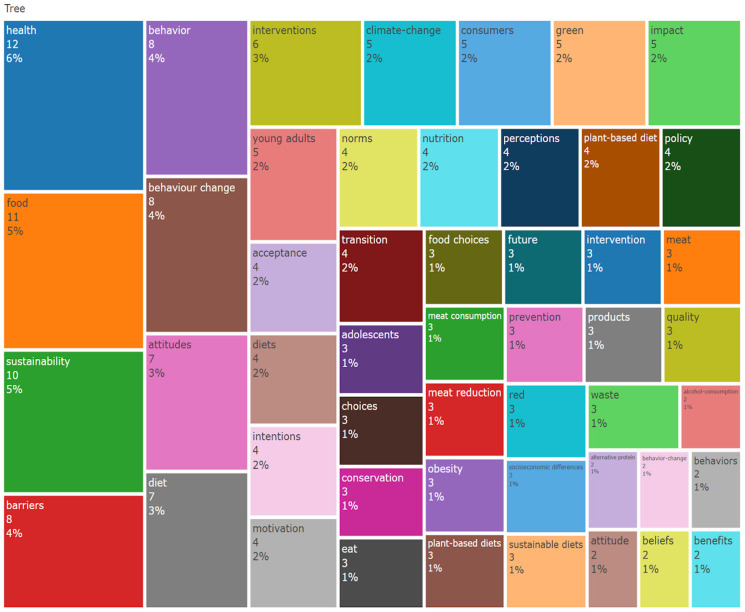
Keywords Tree Map.

To provide a comprehensive overview, the keyword analysis was conducted in two stages. Figure 8a presents raw keyword frequencies to verify the effectiveness of the search strategy, while Figure 8b shows a refined analysis in which primary search terms were excluded to reveal latent thematic clusters and emerging research directions, such as sustainability barriers and dietary transitions. Keyword frequencies from Figure 8b are further quantified using a hierarchical tree map (Figure A2 in Appendix C), illustrating the relative proportion of each term within the dataset (Paul & Criado, 2020).

“Health” emerges as the most prominent keyword (12 occurrences; 6%), occupying a central position in both the word cloud and the treemap, and reflecting its role as the overarching research theme. The analysis reveals that “food” (11 occurrences, 5%) and “sustainability” (10 occurrences, 5%) form a socio-ecological nexus. This cluster suggests how dietary transitions (e.g., “plant-based diet”, “meat reduction”) act as a functional bridge between the environmental dimension (climate change, conservation) and the social and ethical dimension (health, nutrition, and consumer ethics) of SDG 12 (e.g., “plant-based diet”, “meat reduction”) to act as a functional bridge between the environmental dimension (climate change, conservation) and the social and ethical dimension (health, nutrition, and consumer ethics) of SDG 12. A second tier of keywords reflects a strong emphasis on behavioral mechanisms, with “behavior”, “behavior change”, and “barriers” (eight occurrences each; 4%), alongside “attitudes” and “diet” (seven occurrences each; 3%), suggesting the centrality of beliefs and everyday eating practices. The third tier captures intervention-oriented and contextual themes, including “interventions” (six occurrences, 3%), “climate-change” (five occurrences, 2%) and population-focused terms such as “young adults” and “consumers” (five occurrences each, 2%) suggesting the potential for long-term lifestyle modification. Furthermore, emerging research frontiers are reflected in keywords such as “intentions”, “norms”, “perceptions”, “policy”, and “plant-based diet”, each accounting for about 2% of the dataset.

### 3.2. Science Mapping

#### 3.2.1. Co-Authorship Analysis

**Figure 9:**
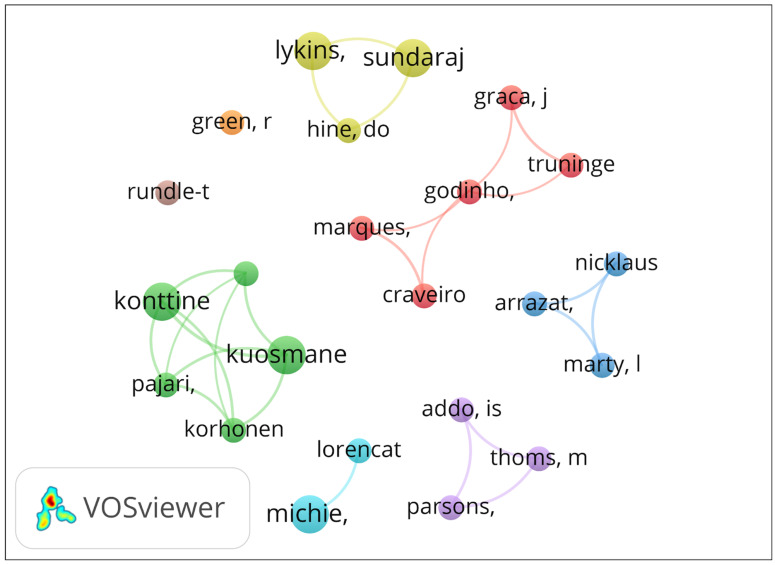
Co-authorship Clusters.

A co-authorship analysis reveals the structure of research collaboration by integrating the authors’ academic backgrounds, thematic focus, and geographical affiliation (Passas, 2024; Perianes Rodríguez et al., 2010). As shown in Figure 9, the network comprises eight clusters and exhibits a largely decentralized social structure with high thematic fragmentation among the 23 core researchers. The most cohesive collaborative hub appears in cluster 2 (green), led by Konttinen and Kuosmanen, which shows the strongest internal integration (total link strength of 9). By contrast, cluster 6 (light blue), comprising Michie and Lorencatto, represents the intellectual foundation of the field; despite a low total link strength (2), this group displays the highest scholarly impact. Additional specialized clusters include cluster 1 (red), led by Craveiro, Godinho, and Marques; cluster 3 (purple), (Arrazat, Marty, and Nicklaus), cluster 5 (Addo, Parsons, and Thoms) and cluster 4 (green), involving Lykins, Hine, and Sundaraj. The co-authorship network is characterized by minimal inter-cluster connectivity and multiple isolated nodes, with the largest connected sub-network comprising five authors. This indicates that the research field remains organized into discrete regional or thematic groups, rather than a globally integrated collaborative system.

#### 3.2.2. Co-Citation Analysis

**Figure 10:**
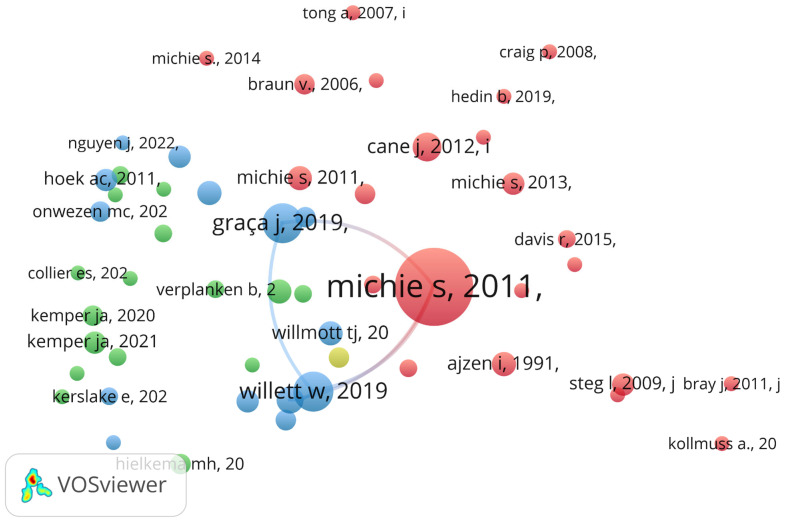
Co-citation Network Analysis (cited references).

The co-citation analysis maps the intellectual structure of the field by examining the relationships among the references cited within the sample documents, with two sources considered co-cited when they appear together in a third document (Aria & Cuccurullo, 2017). Applying a minimum threshold of four citations per reference yielded a core set of 53 influential sources (Figure 10 and Table A3, Appendix D). The resulting network comprises four thematic clusters and 777 significant links, providing a clear representation of the field’s conceptual structure and dominant theoretical trajectories while excluding peripheral or low-impact citations.

The red cluster (theoretical hub) represents the core knowledge base of the field, centered on the COM-B model and BCW (Cane et al., 2012; Michie et al., 2011) The high link density within this cluster indicates a strong theoretical integration and close conceptual alignment. The blue cluster (sustainability transition) is anchored by (Graça et al., 2023; Willett et al., 2019), focusing on the shift toward a plant-based diet within a global food system. The green cluster (consumer psychology) focuses on specific dimensions of consumer behavior, particularly on meat reduction (Kemper, 2020), and meat substitutes (Kerslake et al., 2022), mapping the psychological dimensions of the meat-diet domain. Finally, the yellow cluster (COM-B application led by Sijtsema et al., 2021) specifically explores the capabilities and opportunities of flexitarians, linking theoretical constructs directly to consumer practice.

#### 3.2.3. Co-Word Analysis

**Figure A3:**
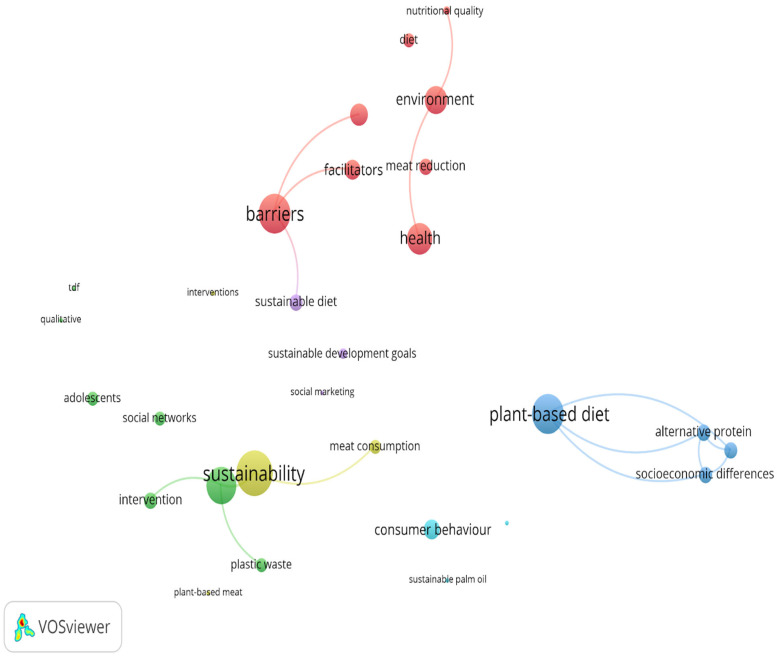
Keywords Network Visualization.

A keyword co-occurrence analysis extends beyond frequency counts to capture the structural relationships among concepts, revealing how behavioral frameworks are operationalized across consumption domains (Figure A3, Appendix D). After data cleaning and term consolidation, the network comprised 29 keywords organized into six clusters (73 links; total link strength = 90). High-centrality nodes—food, health, attitudes, and behavior—anchor the network, linking behavioral mechanisms with dietary practices and sustainability contexts. While sustainability functions as an important connector, empirical activity is most densely concentrated around health-related behaviors, demographic-specific barriers (particularly among young adults), and intervention design. The relative isolation of the plant-based diet cluster suggests a specialized but weakly integrated research niche.

**Figure 11:**
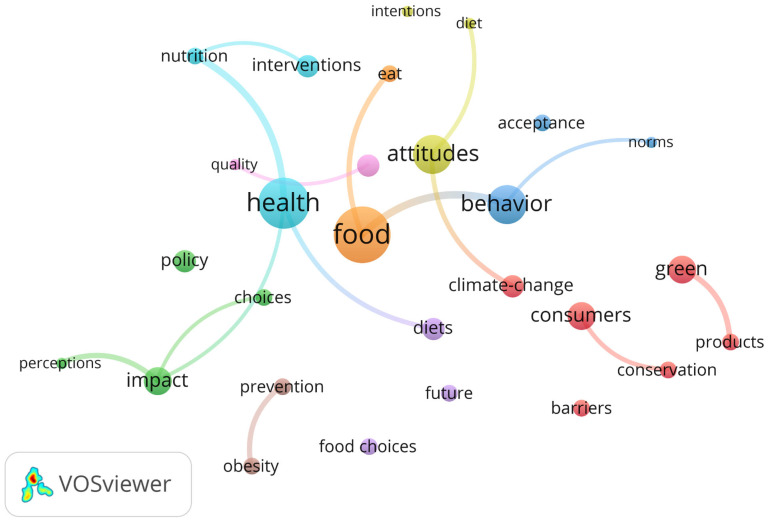
Key Words Plus Co-occurrence Network Visualization.

**Figure 12:**
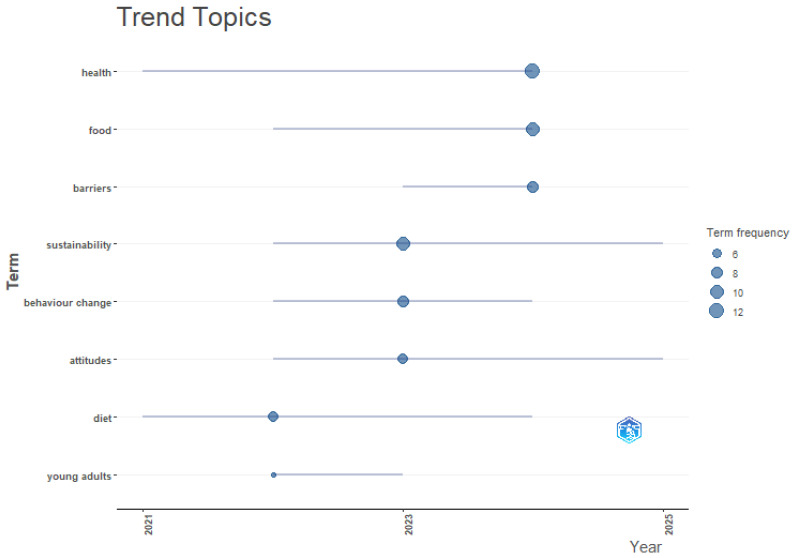
Trend Topics.

To reduce the subjectivity associated with author-defined keywords, a complementary keyword plus co-occurrence analysis was conducted (Figure 11). The resulting network (28 nodes; 113 links; TLS = 55.50) confirms the same intellectual core identified in the author keyword analysis, while further refining the thematic structure through an algorithmic extraction of concepts from the cited references. The analysis identifies food, health, attitudes, and behavior as central mediating nodes and delineates nine clusters spanning environmental drivers (e.g., climate change, green), psychological mechanisms (e.g., intentions, perceptions), and applied intervention domains (e.g., nutrition, policy). This pattern is further reflected in the Trend Topics analysis (Figure 12).

The analysis shows the coexistence of long-standing thematic anchors (health, food, diet), alongside emerging interests in behavior change, attitudes, and sustainability, with the peak intensity observed between 2023 and 2025. Terms such as “young adults” and “barriers” display shorter but highly concentrated periods of prominence, reflecting their relevance in applied, intervention-oriented research. The shift from broad dietary descriptors toward specific psychological constructs, such as “intentions” and “perceptions”, suggests a move from descriptive analyses toward diagnosing the underlying drivers of the intent–behavior gap-. This transition highlights the need for a deeper qualitative and structural examination of how these constructs are operationalized across diverse socio-economic contexts.

## 4. Content Analysis

The content analysis complements the bibliometric findings by examining how responsible consumption is addressed across the selected literature. Bibliometric patterns indicate a strong concentration on individual level behavioral determinants, particularly attitudes, intentions, and health-related drivers, alongside a comparatively peripheral treatment of policy and systemic factors. Building on these insights, the content analysis systematically assessed three interrelated dimensions: the analytical level at which behavioral influences are analyzed (micro, meso, or macro), the primary SDG 12 target addressed by each study, and the geographical context and associated country income group. A detailed overview of these dimensions is provided in Table A4 (Appendix E).

Co-occurrence networks were contextualized within the global policy agenda through a systematic interpretive SDG overlay grounded in the official targets and indicators of UN Resolution 71/313. High-centrality nodes identified in the VOSviewer analysis were manually mapped onto the semantic fields of SDG 12, as detailed in the thematic mapping matrix (Table A5, Appendix E). Although SDG 12.6 is formally classified as a production-side target, prior research conceptualizes organizations as consumers of sustainability practices, information, and institutional norms (Jackson, 2005; Spaargaren, 2011). Accordingly, this study interprets SDG 12.6 as a meso-level consumption target, capturing the behavioral uptake and institutionalization of sustainability practices within organizational contexts.

The analysis confirms that the field’s intellectual structure is strongly oriented toward lifestyle-based behavioral change under SDG Target 12.8, as reflected by high-centrality nodes such as “food”, “health”, and “behaviour”, while systemic targets like sustainable procurement (12.7) remain peripheral. To examine this orientation systematically, each of the 56 COM-B-based studies were coded according to the specific SDG 12 targets addressed, using a structured, theory-driven protocol. The protocol was designed to resolve the conceptual ambiguity inherent in SDG 12, where the boundary between production and consumption is often blurred (e.g., circular economy, food systems, waste flows). The aim was to isolate responsible consumption within the broader SDG 12 agenda and to identify the behavioral pathways most consistently supported by COM-B research. An SDG target was coded only when studies explicitly examined consumer behavior, household practices, lifestyle change, public procurement as institutional consumption, or behavioral responses to policy instruments (e.g., price signals, awareness campaigns) (Table A5, Appendix E). This approach enabled the identification of the dominant behavioral pathways within SDG 12 and revealed that most COM-B applications align with Target 12.8 (awareness and sustainable lifestyles), with more limited engagement with hybrid targets such as 12.3 (food waste) and 12.5 (waste reduction and recycling).

Six specific SDG 12 targets were identified across the studies applying the COM-B model. The total number of targets mentions (n = 70) exceeds the total number of articles (N = 56) due to the application of a multinodal coding approach, reflecting the inherently multifaceted nature of responsible consumption. For example, educational activities (SDG 12.8) often serve as a tool for achieving waste-prevention goals (SDG 12.3 or SDG 12.5). Accordingly, 20% of the articles exhibit a complex, multi-target orientation with respect to SDG 12 (e.g., (Galibourg et al., 2024; Sundaraja et al., 2024)). The dominance of SDG 12.8 directly mirrors the keyword clusters identified in the bibliometric analysis, where health, food, behavior, attitudes, and diet form the central thematic core. These clusters align with lifestyle-oriented behavioral change, which constitutes the conceptual foundation of SDG 12.8. Similarly, the co-citation network is anchored in the Behavior Change Wheel (Michie et al., 2011) and the dietary sustainability literature (Willett et al., 2019), both of which prioritize individual-level behavioral mechanisms. This intellectual structure is reflected in the overwhelming prevalence of SDG 12.8 in the empirical corpus. By contrast, targets related to waste reduction (12.5) and food waste (12.3) appear only in peripheral clusters of the keyword network (e.g., waste, reuse, conservation), consistent with their limited representation in the sample.

Translating behavioral theory (particularly insights into the factors shaping individual decision-making) into practical, effective, and scalable interventions requires a clear systematization of the levels at which these influences operate (Table A4, Appendix E). Accordingly, the analytical framework adopted a multi-level perspective, examining research objects across three interconnected scales: the micro-level, focusing on individual subjects, cognitive processes, motivations, and behavioral patterns; the meso-level, which captures institutional and organizational contexts, such as professional networks, universities, healthcare institutions, and community programs; and the macro-level, which addresses systemic dynamics, such as government regulations, national policy frameworks, and broader socio-cultural transformations.

Given the complexity of the analyzed articles, traditional unitary coding was expanded through a hybrid additive approach that explicitly captures cross-level interactions: micro–meso linkages where organizational environments shape individual behavior; micro–macro connections linking personal practices to policy instruments or global objectives; and fully multi-level analyses integrating all three scales within a systems-oriented framework. This distinction is critical for intervention design, as it informs the selection of behavior-change techniques and tools ranging from individual-level learning and motivational techniques to organizational practices and regulatory instruments, thereby supporting behavior change across multiple levels of society.

Applications of the COM-B model are strongly skewed toward the micro-level, with 50% of the studies (n = 28) focusing exclusively on individual psychological determinants (Allenden et al., 2026; Ježovit & Lučić, 2025). Hybrid coding reveals meaningful cross-level engagement, with 26.8% of the studies (n = 15) addressing micro–meso interactions related to organizational or contextual influences (Guzmics & Kutzner, 2025; Mezzacapo et al., 2025), while 12.5% of the studies (n = 7) linked individual behavior to macro-level dynamics such as global trends or policy contexts (Allison et al., 2022; Van Hau et al., 2025). A full multilevel analysis, integrating the micro-, meso-, and macro-scale, was observed in only 8.9% (n = 5) of the publications (Chad, 2023; Craveiro et al., 2021; Graça et al., 2023; Gupta et al., 2025; Sundaraja et al., 2023).

Notably, no studies focused solely on meso- or macro-levels (n = 0) without an individual behavioral component, and only one study (n = 1) examined the meso–macro nexus in isolation. This distribution is consistent with the field’s intellectual core, as reflected in the dominant journals (Appetite, Food Quality and Preference, Sustainability), the co-citation core (Behavioral Change Wheel/Theoretical Domains Framework), and the keyword core (attitudes, behavior, diet). These patterns suggest that micro-level dominance is a structural characteristic of COM-B applications in responsible consumption research. Meso-level analyses are typically situated in collective food contexts, educational settings, or household waste practices, while macro-level engagement remains largely confined to systematic reviews, modeling studies, and policy-adjacent research.

The geographical distribution of the 56 studies is highly concentrated. Australia accounts for 10 studies (17.9%); the United Kingdom for 9 studies (16.1%); Finland, France, and the Netherlands collectively for 7 studies (12.5%. Upper-middle-income countries contributed three studies (5.4%), lower-middle-income countries contributed one study (1.8%), and low-income/LMIC clusters contributed one study (1.8%). An additional nine studies (16.1%) were not region-specific, including systematic reviews and meta-analyses. This distribution closely mirrors the bibliometric collaboration network, where Anglophone and Northern European countries form the structural core of the field. These regions also dominate the institutional landscape, with Utrecht University, the University of Helsinki, and Australian universities among the most productive. The regional concentration explains the thematic focus on dietary transitions, behavioral determinants, and lifestyle change—all central to SDG 12.8.

Using the World Bank income classifications, 75% of the analyzed studies (n = 42) were conducted in high-income countries, with only limited representation from upper-middle-income (5.4%), lower-middle-income (1.8%), and low- and middle-income country contexts (1.8%) (Table A4, Appendix E). An additional 16.1% of the publications were not region-specific, consisting of systematic reviews and meta-analyses. Geographically, the empirical research is heavily concentrated in the United Kingdom and Australia, which together account for nearly 40% of all studies, followed by Northern European countries and the Netherlands. This spatial concentration closely aligns with the co-authorship and institutional networks identified in the bibliometric analysis and provides a structural explanation for the predominance of lifestyle-oriented SDG 12.8 research and the prevailing focus on micro-level COM-B applications.

Across all analytical dimensions, the content analysis closely mirrors the structural characteristics identified through bibliometric mapping. The concentration of publications within a small set of core journals, consistent with Bradford’s Law, helps explain the thematic dominance of dietary behavior and sustainability lifestyle research. The co-citation structure reinforces a strong micro-level orientation and behavioral focus, while the configuration of keyword clusters accounts for the prevalence of studies aligned with SDG 12.8 (“By 2030, ensure that people everywhere have the relevant information and awareness for sustainable development and lifestyles in harmony with nature”). At the same time, the co-authorship network reveals regional and thematic areas of expertise, predominantly located in high-income contexts. These geographical and income level patterns help explain the limited engagement with waste-related and policy-oriented SDG targets in the literature. Finally, the predominance of specific document types contributes to the methodological homogeneity and constrains the coverage of macro-level analysis.

These patterns suggest that the thematic and analytical characteristics of COM-B applications in responsible consumption are structurally embedded in the intellectual, geographical, and institutional configuration of the field.

The SDG 12 target-indicator mapping (Table A5, Appendix E) shows that, despite the theoretical versatility of the COM-B framework, its empirical application within responsible consumption research remains largely confined to behavioral explanation rather than policy diagnosis. The concentration on Target 12.8 reflects a consistent operationalization of the COM-B model at the micro-level, where motivation and, to a lesser extent, psychological capability are examined through attitudes, intentions, and awareness-raising interventions. By contrast, SDG 12 targets that require structural, regulatory, or economic transformation (e.g., 12.1, 12.2, 12.7, 12.8) are either weakly represented or entirely absent, indicating that opportunity is rarely conceptualized as an institutional or policy-driven construct. Even where policy-adjacent targets such as 12.6 and 12.7 are addressed, the COM-B model is primarily used to assess acceptance, norms, or behavioral compliance rather than to diagnose how governance arrangements, procurement systems, or market incentives shape behavioral opportunity at meso- or macro-levels. This may point to a systematic underutilization of the COM-B model as a policy diagnostic tool, reinforcing its current positioning as a framework for individual-level behavioral analysis rather than integrated consumption–policy design.

## 5. Discussion, Conclusion and Limitations

### 5.1. Discussion

#### 5.1.1. Thematic Consolidation and Intellectual Lineage

The synthesis of bibliometric and content-analytic findings points to a research domain undergoing rapid consolidation. The cumulative growth of leading journals suggests an “active expansion phase”, characterized by a shift from descriptive accounts of consumption to mechanism-based behavioral diagnostics. At the center of this transition, Michie’s Behaviour Change Wheel and the COM-B model serve as a methodological bridge connecting foundational implementation science with planetary health imperatives. This pattern may signal a conceptual movement from Ajzen’s theory of planned behavior (Ajzen, 1991) toward a more diagnostic, intervention-oriented approach. Within the context of our COM-B framework analysis, this shift is driven by the need to move beyond predicting intentions to identifying the specific behavioral drivers that serve as a functional base for designing effective sustainability interventions (Han, 2021; Hosta & Zabkar, 2021). By addressing the “intention-behavior gap” (Sheeran & Webb, 2016), such diagnostic frameworks significantly increase predictive power (Hosta & Zabkar, 2021) and therefore the practical relevance of the results.

#### 5.1.2. Structural Asymmetries: Geography, Institutions, and Authorship

The geographical configuration of the field appears to function as a structural factor shaping its conceptual orientation. The dominance of the UK, Australia, and the Netherlands in the observed network is consistent with Alatas’ (2003) theory of academic dependency, creating a relatively homogeneous epistemic space, in which research agendas are defined along a Global North axis. While this facilitates efficient knowledge diffusion, it may also contribute to a pronounced “WEIRD” bias (Shepherd et al., 2025), favoring Western, educated, industrialized, rich, and democratic contexts. Consequently, physical opportunity barriers unique to emerging economies, such as infrastructure deficits or regulatory gaps, appear to remain comparatively underexplored in the current evidence base. Notably, an analysis of the QS World University Rankings 2026 suggests a potential divergence between domain-specific productivity and global institutional prestige. This pattern provides external validation of the field’s structural characteristics. Productivity leaders such as the University of Helsinki (QS 116) and the University of New England (QS 1001–1200) outperform many top 100 institutions, suggesting that research leadership in responsible consumption may be driven more by specialized departmental expertise than by overall institutional prestige. This decentralized expertise is further characterized by a “transient authorship” pattern, consistent with Lotka’s law of scientific productivity, where 92.19% of the authors contribute a single publication. This indicates that the COM-B model is frequently used as an episodic thematic extension rather than a sustained research focus, reinforcing the field’s emergent and exploratory status.

#### 5.1.3. Micro-Level Focus in Responsible Consumption Research

A central pattern in the literature is the strong dominance of micro-level analyses. Despite its theoretical capacity to address systemic issues, the COM-B model is primarily operationalized to diagnose individual psychological determinants, such as attitudes and motivation, a tendency likely rooted in the framework’s origins in clinical health settings. Although the COM-B model was originally developed to explain individual behavior, its explicit incorporation of social and physical opportunity allows for analysis at institutional and policy-relevant levels.

The scarcity of meso- (organizational) and macro- (policy) level studies may constrain the field’s ability to address the structural levers essential for a circular economy, such as procurement systems and price distortions. This imbalance is particularly visible in the current evidence base in the focus on SDG Target 12.8, while other targets receive disproportionately less attention. These findings suggest that the COM-B model is currently treated as a behavioral explanation model, rather than a policy diagnostic framework. This narrowing of analytical scope may constrain its broader systemic ambition, particularly regarding the “opportunity” component, which remains under-theorized at institutional and regulatory levels

### 5.2. Limitations

While this study provides a comprehensive synthesis of COM-B applications in the research, it was confined to the Web of Science database to ensure metadata consistency. Although the WoS covers high-impact journals, it may exclude relevant studies indexed in other databases, as well as grey literature which often contains practical policy insights. Second, bibliometric metrics, such as citation counts, serve as proxies for scholarly influence but do not directly capture real-world behavioral change or tangible sustainability outcomes. Finally, the content analysis relied on reported data in published articles, and may therefore reflect “publication bias”, as successful interventions are more likely to be documented than null or inconclusive results (Dowdy et al., 2022). As Tincani and Travers (2019) argue, such bias may “unduly overestimate” the effectiveness of the COM-B framework, obscuring contexts where the model fails to bridge the gap between intentions and behavior. Our findings may therefore overstate the universal applicability of certain COM-B drivers in the contexts of UN SDG 12. However, according to Ramos (2026), journal bias can be a useful filter. In fields where true behavioral effects are difficult to achieve, statistically significant results may be more likely to reflect meaningful changes.

## 6. Conclusions

This study shows that, although the applications of the COM-B model to responsible consumption are expanding rapidly, the field remains structurally imbalanced. The COM-B model has proven highly effective in diagnosing individual-level behaviors aligned with SDG 12.8, particularly in high-income contexts, yet its capacity to inform meso- and macro-level transformations remains largely underutilized. By integrating bibliometric mapping with theory-driven content analysis, this study clarifies not only where the COM-B model is applied within responsible consumption research, but how and at which systemic levels its diagnostic capacity remains underexploited.

To advance the field, future research must move beyond diagnosing individual intentions toward the experimental evolution of real-world environmental restructuring. Priority should be given to multi-level study designs that integrate behavioral diagnostics with circular economy agendas and extend empirical inquiry to diverse economic contexts beyond Global North contexts. By bridging individual psychology with structural opportunity, the scientific community will be better positioned to develop robust, system-wide strategies capable of addressing the full spectrum of SDG 12 targets. From an SDG 12 perspective, the current imbalance suggests that COM-B applications primarily support behavioral awareness (Target 12.8), while offering limited diagnostic insight into consumption systems governed by institutional, economic, and regulatory dynamics.

## Figures and Tables

**Table 1:** Descriptive Statistics.

Database	No. of Articles	Time	Document Avg. Age	No. of Publishing Sources	Average Citation per Article	Highest No. of Citations per Article
WoS	56	2018–2026	2.16	37	20.62	323

**Table 2:** Institution’s performance.

Organization	Country	No. of Articles	QS Rank (2026) *
University of Helsinki	Finland	15	116
University of New England	Australia	12	1001–1200
Griffith University	Australia	6	268
Loughborough University	UK	6	225
University of Lisbon	Portugal	6	230
Utrecht University	Netherlands	6	103
National University of Ireland Galway	Ireland	5	284
Brunel University of London	UK	4	385
Flinders University	Australia	4	387
Oxford Brookes University	UK	4	374

* QS Ranking—QS University Ranking 2026. (Top Universities. (2025, June 19). QS World University Rankings 2026: Top global universities. Top Universities. https://www.topuniversities.com/world-university-rankings, accessed on 16 January 2026).

**Table 3:** Contributing journals as per number of articles and citation counts.

Journals as per Number of Articles		Journals as per Number of Citations	
Journal	No. of Articles	Journal	No. of Citations
Appetite	9	Trends in Food Science & Technology	328
Food Quality and Preference	4	Appetite	137
Sustainability	4	Implementation Science	94
Frontiers in Nutrition	2	Sustainability	84
Journal of Cleaner Production	2	Sustainable Production and Consumption	79
Public Health Nutrition	2	Journal of Cleaner Production	47
Sustainable Futures	2	Water Resources Research	46
Sustainable Production and Consumption	2	Pilot And Feasibility Studies	41
Australasian Marketing Journal	1	Preventive Medicine Reports	37

**Table 4:** The Cumulative Growth of the Most Productive Journals.

Journal	2018	2019	2020	2021	2022	2023	2024	2025	2026
Appetite	0	1	1	2	2	5	6	9	9
Food Quality and Preference	0	0	0	0	1	2	2	3	4
Sustainability	0	0	0	2	2	2	3	4	4
Frontiers in Nutrition	0	0	0	0	0	1	2	2	2
Journal of Cleaner Production	0	0	0	0	2	2	2	2	2
Public Health Nutrition	0	0	0	1	1	1	2	2	2
Sustainable Futures	0	0	0	0	0	0	0	2	2
Sustainable Production and Consumption	0	0	0	0	1	2	2	2	2

**Figure A1:**
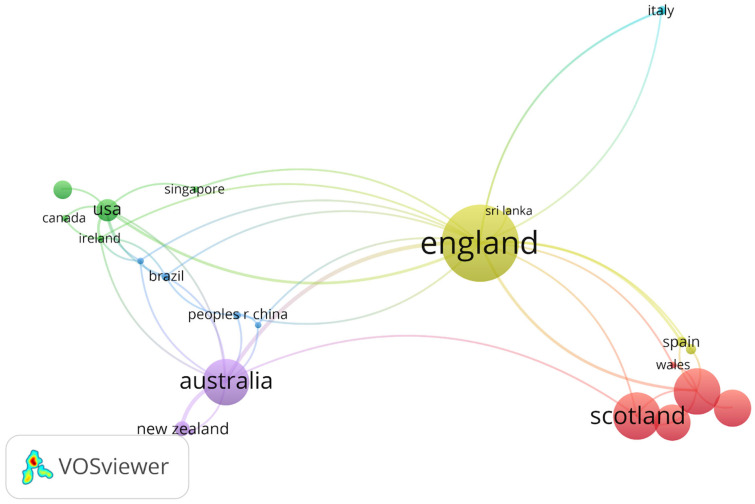
VOSviewer Topological Analysis.

## Data Availability

No new data were created or analyzed in this study. The dataset collected from Web of Science and analyzed in this research is available on request.

## Abbreviations

The following abbreviations are used in this manuscript:

BCW	Behavior Change Wheel
COM-B	Capability–Opportunity–Motivation–Behavior
SDG	Sustainable Development Goal
WoS	Web of Science

## Appendix B. Author’s Performance

**Table A1:** Scientific Productivity Distribution—Lotka’s Law.

Written Documents	No. of Authors	Observed Frequency (%)	Theoretical Frequency (Lotka’s %)
1	248	92.19	73.47
2	16	5.95	18.37
3	5	1.86	8.16

**Table A2:** Author’s Performance.

Author	First Publication Year	No. of Articles	Fractionalized Articles	Total Citations (TC)	h-Index	Affiliation
GRACA JOAO	2019	2	0.476	348	2	University of Groningen, Netherlands
MICHIE SUSAN	2018	3	0.560	105	3	UCL, England
ADDO ISAAC B	2018	2	0.667	81	2	University of New England, Australia
LORENCATTO FABIANA	2021	2	0.417	87	2	UCL, England
CRAVEIRO DANIELA	2021	2	0.25	21	2	University of Lisbon, Portugal
GODINHO CRISTINA	2021	2	0.310	25	2	NOVA University of Lisbon, Portugal
HINE DONALD W	2021	2	0.533	27	2	Canterbury University, New Zealand
GREEN ROSEMARY	2024	2	0.75	42	2	London School of Hygiene & Tropical Medicine
KUOSMANEN SINI	2023	3	0.643	17	2	University of Helsinki, Finland
KONTTINEN HANNA	2023	3	0.393	17	2	University of Helsinki, Finland
LYKINS AMY D	2021	3	0.733	27	1	University of New England, Australia
SUNDARAJA C.S.	2021	3	0.783	28	2	University of New England, Australia

## Appendix D. Co-Word Analysis

**Table A3:** Bibliometric and conceptual profiles of the most influential publications within the Co-citation clusters.

Cluster	Authors	Title	Year	TLS	Author Keywords	Keywords Plus	Citations	Web of Science Categories	Journal
Red	Michie, S; van Stralen, MM; West, R	The behaviour change wheel: a new method for characterising and designing behaviour change interventions	2011	238	-	Implementation, health, psychology	43	Health Care Sciences & Services; Health Policy & Services	Implementation science
Red	Cane, J; O’Connor, D; Michie, S	Validation of the theoretical domains framework for use in behaviour change and implementation research	2012	54	Theoretical domains framework, behaviour change implementation, validation theory	Low-back-pain, health, behavior knowledge, interventions, guideline, classification, impairment, prevention, smoking, trial	10	Health Care Sciences & Services; Health Policy & Services	Implementation science
Blue	Graça, J; Godinho, CA; Truninger, M	Reducing meat consumption and following plant-based diets: Current evidence and future directions to inform integrated transitions	2019	178	Plant-based diets; meat substitution; meat consumption; sustainability; health	Sustainable food choices, behavior change wheel, consumer attitudes; climate-change; red meat; conscientious omnivores; principal-components; protein consumption; moral disengagement; mediating role	16	Food Science & Technology	Trends in food science & technology
Blue	Willett, W; Rockstrom, J; Loken, B; Springmann, M; Lang, T; Vermeulen, S; Garnett, T; Tilman, D; DeClerck, F; Wood, A; Jonell, M; Clark, M; Gordon, LJ; Fanzo, J; Hawkes, C; Zurayk, R; Rivera, JA; De Vries, W; Sibanda, LM; Afshin, A; Chaudhary, A; Herrero, M; Agustina, R; Branca, F; Lartey, A; Fan, SG; Crona, B; Fox, E; Bignet, V; Troell, M; Lindahl, T; Singh, S; Cornell, SE; Reddy, KS; Narain, S; Nishtar, S; Murray, CJL	Food in the Anthropocene: the EAT-Lancet Commission on healthy diets from sustainable food systems	2019	164	-	Coronary-heart-disease; greenhouse-gas emissions; trans-fatty-acids; Sub-Saharan Africa; cardiovascular-disease; meat consumption; carbohydrate intake; nut consumption; breast-cancer; agricultural intensification	16	Medicine, General & Internal	Lancet
Green	Kemper, JA	Motivations, barriers, and strategies for meat reduction at different family lifecycle stages	2020	84	Flexitarian; meat reduction; meat consumption; focus groups	Protein consumption; climate-change; vegetarians attitudes; consumers; environment; beliefs; health; perceptions; transitions	6	Behavioral Sciences, Nutrition & Dietetics	Appetite
Green	Kerslake, E; Kemper, JA; Conroy, D	What’s your beef with meat substitutes? Exploring barriers and facilitators for meat substitutes in omnivores, vegetarians, and vegans	2022	83	Meat substitutes; meat reduction; vegan; vegetarian; flexitarian	Consumer preferences; focus groups; food choice; consumption attitudes; product; diet; sustainability; behavior; insects	5	Behavioral Sciences, Nutrition & Dietetics	Appetite
Yellow	Sijtsema, SJ; Dagevos, H; Nassar, G; de Winter, MV; Snoek, HM	Capabilities and Opportunities of Flexitarians to Become Food Innovators for a Healthy Planet: Two Explorative Studies	2021	55	flexitarianism; COM-B model; plant-based; meat substitutes; focus group discussion; food practices	Meat consumption	6	Green & Sustainable Science & Technology; Environmental Sciences; Environmental Studies	Sustainability

## Appendix E. Content Analysis

**Table A4:** Systematic content analysis and coding matrix of the analyzed corpus (n = 56).

ID	Surname, Year	Dominant Level	Primary Target (12.x)	Source	Study Region	Income Group
A01	Ježovit, M., & Lučić, A. (2025)	Micro	12.8	Journal of Marketing Analytics	Croatia	High income
A02	McBey, D., Sanchez, G. M., Horgan, G., Macdiarmid, J. I., & McCormick, B. J. J. (2026).	Micro + Macro	12.8	Food Quality and Preference	Scotland	High income
A03	Allenden, N., Lykins, A. D., Phillips, K. L., & Cosh, S. M. (2026)	Micro	12.8	Journal of Health Psychology	Australia	High income
A04	Van Hau, P., Robertson, K., Thyne, M., Hamlin, R., & Rundle-Thiele, S. (2025)	Micro + Macro	12.5	Sustainable Futures	Not region-specific	Not specified
A05	Guzmics, D., & Kutzner, F. (2025)	Micro + Meso	12.8	Transportation Research	Germany/Austria	High income
A06	Lavelle, F., George, V. C., Mckernan, C., Martins, C. A., Shrewsbury, V. A., Wolfson, J. A., Taylor, R. M., Duncanson, K., Elliott, C., & Collins, C. E. (2025)	Micro	12.8	Appetite	UK & Ireland	High income
A07	Lee, D., Wan, C., Isbanner, S., Rundle-Thiele, S., & Fung, Y.-N. (2025)	Micro	12.8	Sustainable Futures	China	Upper middle income
A08	Gupta, K., Seddighi, M. J., Davies, E. L., Thondre, P. S., & Samangooei, M. (2025)	Micro + Meso + Macro	12.8	Sustainability	UK	High income
A09	Kuosmanen, S., Koponen, S., Konttinen, H., & Niva, M. (2025)	Micro	12.8	Future Foods	Nordic countries	High income
A10	Mezzacapo, U., Voltolina, D., Gencarelli, C. N., Esposito, G., Mondini, A., Salvati, P., Tondini, S., Carlone, T., Sarretta, A., Galizia, A., Sterlacchini, S., & Marchesini, I. (2025)	Micro + Meso	12.8	International Journal of Disaster Risk Reduction	Italy	High income
A11	Anant, N., Pillay, A., Juraimi, S. A., Sheen, F., Fogel, A., Chong, M. F.-F., Smith, B. P. C., & Pink, A. E. (2025)	Micro	12.8	Appetite	Singapore	High income
A12	Arrazat, L., Teil, F., Nicklaus, S., & Marty, L. (2025).	Micro + Meso	12.8	Appetite	France	High income
A13	Kuosmanen, S., Korhonen, K., Pajari, A.-M., & Konttinen, H. (2025).	Micro	12.8	Food Quality and Preference	Finland	High income
A14	George, L., & Davison, J. (2024)	Micro	12.8	Public Health Nutrition	UK	High income
A15	Addo, I. B., Thoms, M. C., & Parsons, M. (2018)	Micro	12.8	Water	Australia	High income
A16	Addo, I. B., Thoms, M. C., & Parsons, M. (2018)	Micro	12.8	Water Resources Research	Not region-specific	Not specified
A17	Allison, A. L., Baird, H. M., Lorencatto, F., Webb, T. L., & Michie, S. (2022)	Micro + Macro	12.5 [Primary]; 12.8 [Partial]	Journal of Cleaner Production	Not region-specific	Not specified
A18	Allison, A. L., Lorencatto, F., Michie, S., & Miodownik, M. (2021)	Micro + Meso	12.6 [Primary]; 12.5 [Partial]	Sustainability	UK	High income
A19	Arrazat, L., Nicklaus, S., de Lauzon-Guillain, B., & Marty, L. (2024)	Micro + Meso	12.8	Appetite	France	High income
A20	Beck, A. L., Iturralde, E., Haya-Fisher, J., Kim, S., Keeton, V., & Fernandez, A. (2019)	Micro + Meso	12.8	Appetite	USA	High income
A21	Bryant, C., Ross, E., & Flores, C. (2023)	Micro	12.8	Food Quality and Preference	UK	High income
A22	Ceballos, A. M. G., & Antonopoulou, V. (2024)	Micro + Meso	12.3, 12.5	Current Psychology	Colombia	Upper middle income
A23	Chad, P. (2023)	Micro + Meso + Macro	12.5 [Primary]; 12.8 [Partial]	Australasian Marketing Journal	Australia	High income
A24	Cradock, K. A., Quinlan, L. R., Finucane, F. M., Gainforth, H. L., Ginis, K. A. M., Sanders, E. B.-N., & OLaighin, G. (2022)	Micro	12.8	Sensors	Ireland	High income
A25	Craveiro, D., Marques, S., Bell, R., Khan, M., Godinho, C., & Peixeiro, F. (2021)	Micro + Meso + Macro	12.8	Public Health Nutrition	Portugal	High income
A26	Craveiro, D., Marques, S., Zverinova, I., Maca, V., Scasny, M., Chiabai, A., Suarez, C., Martinez-Juarez, P., Garcia de Jalon, S., Quiroga, S., & Taylor, T. (2021)	Micro + Macro	12.8	Appetite	Czech Republic, Latvia, Portugal, Spain, and the UK	High income
A27	da Veiga, C. P., Moreira, M. N. B., da Veiga, C. R. P., Souza, A., & Su, Z. (2023)	Micro	12.8	Foods	Brazil	Upper middle income
A28	Davis, N., Dermody, B., Koetse, M., & van Voorn, G. A. K. (2024)	Micro + Macro	12.8	JASSS-The Journal of Artificial Societies and Social Simulation	Netherlands	High income
A29	Fechner, D., Gruen, B., & Dolnicar, S. (2025)	Micro + Meso	12.8	Journal of Sustainable Tourism	Australia	High income
A30	Fernandez-Alvarez, M. del M., Cuesta, M., Cachero-Rodriguez, J., Gardner, B., Lana, A., & Martin-Payo, R. (2025)	Micro	12.5	Waste Management & Research	Spain	High income
A31	Ford, H., Gould, J., Danner, L., Bastian, S. E. P., & Yang, Q. (2023)	Micro	12.8	Appetite	UK	High income
A32	Galibourg, D., Scott, R. E., Gough, K. V., Drechsel, P., & Evans, B. E. (2024)	Meso + Macro	12.8 [Primary]; 12.6, 12.3 [Partial]	Journal of Water Sanitation and Hygiene for Development	Low- and middle-income countries	Low- and middle-income countries
A33	Graça, J., Campos, L., Guedes, D., Roque, L., Brazão, V., Truninger, M., & Godinho, C. (2023)	Micro + Meso	12.8	Appetite	Not region-specific	Not specified
A34	Graça, J., Godinho, C. A., & Truninger, M. (2019)	Micro + Meso + Macro	12.8	Trends in Food Science & Technology	Not region-specific	Not specified
A35	Hall, J., Morton, S., Hall, J., Clarke, D. J., Fitzsimons, C. F., English, C., Forster, A., Mead, G. E., & Lawton, R. (2020)	Micro + Meso	12.8	Pilot and Feasibility Studies	UK (England and Scotland)	High income
A36	Hoang, V., Saviolidis, N. M., Olafsdottir, G., Bogason, S., Hubbard, C., Samoggia, A., Nguyen, V., & Nguyen, D. (2023)	Micro	12.8 [Primary]; 12.6 [Partial]	Sustainable Production and Consumption	Vietnam	Lower middle income
A37	Kuosmanen, S., Niva, M., Pajari, A.-M., Korhonen, K., Muilu, T., & Konttinen, H. (2023)	Micro	12.8	Frontiers in Nutrition	Finland	High income
A38	Maluf, V. A. B., Fabbi, S., Azevedo, C. C., & Carrard, I. (2024)	Micro + Meso	12.8	Journal of Nutritional Science	Switzerland	High income
A39	Manika, D., Iacovidou, E., Canhoto, A., Pei, E., & Mach, K. (2022).	Micro + Meso	12.3	Journal of Cleaner Production	England	High income
A40	Mauch, C. E., Laws, R. A., Prichard, I., Maeder, A. J., Wycherley, T. P., & Golley, R. K. (2021)	Micro	12.8	JMIR mHealth and uHealth	Australia	High income
A41	Naicker, A., Shrestha, A., Joshi, C., Willett, W., & Spiegelman, D. (2021)	Micro+ Meso	12.8 [Primary]; 12.1 [Partial]	Preventive Medicine Reports	Not region-specific	Not specified
A42	Palmer, B., Irwin, C., & Desbrow, B. (2024)	Micro	12.8	Drug and Alcohol Review	Australia	High income
A43	Porter, L., Chater, A. M., Haycraft, E., Farrow, C., & Holley, C. E. (2023)	Micro	12.8 [Primary]; 12.3 [Partial]	Appetite	UK	High income
A44	Ran, Y., Lewis, A. N., Dawkins, E., Grah, R., Vanhuyse, F., Engstrom, E., & Lambe, F. (2022)	Micro	12.8 [Primary]; 12.6 [Partial]	Sustainable Production and Consumption	Sweden	High income
A45	Rickerby, A., & Green, R. (2024)	Micro	12.8	Nutrients	High-income countries	High-income countries
A46	Rosario, F., Santos, M. I., Angus, K., Pas, L., Ribeiro, C., & Fitzgerald, N. (2021).	Micro + Meso	12.1	Implementation Science	Not region-specific	Not specified
A47	Rouf, A., Clayton, S., & Allman-Farinelli, M. (2019)	Micro	12.8	Journal of Human Nutrition and Dietetics,	Australia	High income
A48	Sijtsema, S. J., Dagevos, H., Nassar, G., van Haaster de Winter, M., & Snoek, H. M. (2021)	Micro	12.8	Sustainability	Netherlands	High income
A49	Stevely, A. K., Buykx, P., Brown, J., Beard, E., Michie, S., Meier, P. S., & Holmes, J. (2018)	Micro + Macro	12.1	BMC Public Health	UK	High income
A50	Sundaraja, C. S., Hine, D. W., & Lykins, A. D. (2021)	Micro	12.6 [Primary]; 12.8 [Partial]	PLoS ONE	Australia	High income
A51	Sundaraja, C. S., Hine, D. W., & Thorsteinsson, E. B. (2023)	Micro + Macro	12.8	Australian Journal of Environmental Education	Australia	High income
A52	Sundaraja, C. S., Lykins, A. D., & Hine, D. W. (2024)	Micro + Meso + Macro	12.7, 12.1, 12.6	Frontiers in Nutrition	Not region-specific	Not specified
A53	Van Den Berg, S. W., van den Brink, A. C., Wagemakers, A., & den Broeder, L. (2022)	Micro	12.8	Food Quality and Preference	Netherlands	High income
A54	Van Etten, S., Jansen, L., Bal, M., Dermody, B. J., Muller, E., de Wit, J., & Stok, M. (2023)	Micro + Meso	12.8	BMC Nutrition	Netherlands	High income
A55	Vezovnik, A., & Kamin, T. (2024)	Micro	12.8	Sustainability	Slovenia	High income
A56	Wadi, N. M., Cheikh, K., Keung, Y. W., & Green, R. (2024)	Micro	12.8 [Primary]; 12.3 [Partial]	Lancet Planetary Health	High-income countries	High-income countries

**Table A5:** SDG 12 target and indicator mapping matrix.

Goals and Targets (From the 2030 Agenda for Sustainable Development) *	Official Indicators *	Analytical Focus in This Study (Consumption vs. Production)	Associated Keywords (From VOSviewer Analysis)	Number of Studies Addressing the Target (n)
12.1 Implement the 10-Year Framework of Programmes on Sustainable Consumption and Production Patterns.	12.1.1 Number of countries with sustainable consumption and production (SCP) national action plans or SCP mainstreamed as a priority or a target into national policies.	Both (policy framework for Sustainable Consumption and Production)	policy, interventions, impact, future	4 [partial]
12.2 By 2030, achieve the sustainable management and efficient use of natural resources.	12.2.1 Material footprint, material footprint per capita, and material footprint per GDP. 12.2.2 Domestic material consumption, domestic material consumption per capita, and domestic material consumption per GDP.	Production (resource extraction, material flows)	climate-change, conservation, impact, products, green	Not addressed
12.3 By 2030, halve per capita global food waste at the retail and consumer levels and reduce food losses along production and supply chains, including post-harvest losses.	12.3.1 Global food loss index.	Consumption (retail & household waste) + Production (supply chain losses)	food, diets, food choices, eat, diet, prevention, quality	5 [partial]
12.4 By 2020, achieve the environmentally sound management of chemicals and all wastes throughout their life cycle, in accordance with agreed international frameworks, and significantly reduce their release to air, water and soil in order to minimize their adverse impacts on human health and the environment.	12.4.1 Number of parties to international multilateral environmental agreements on hazardous waste, and other chemicals that meet their commitments and obligations in transmitting information as required by each relevant agreement. 12.4.2 Hazardous waste generated per capita and proportion of hazardous waste treated, by type of treatment.	Production (industrial waste, chemicals)	impact, quality	Not addressed
12.5 By 2030, substantially reduce waste generation through prevention, reduction, recycling and reuse.	12.5.1 National recycling rate, tons of material recycled.	Consumption (household waste) + Production (industrial waste)	prevention, conservation, products, green	6
12.6 Encourage companies, especially large and transnational companies, to adopt sustainable practices and to integrate sustainability information into their reporting cycle.	12.6.1 Number of companies publishing sustainability reports.	Production (corporate sustainability) + Consumption (internal consumption of sustainability practices by employees)	policy, behavior, norms, acceptance, interventions, impact	6
12.7 Promote public procurement practices that are sustainable, in accordance with national policies and priorities.	12.7.1 Number of countries implementing sustainable public procurement policies and action plans.	Production (procurement systems) + Consumption (government as consumer)	policy, products, green, impact	1
12.8 By 2030, ensure that people everywhere have the relevant information and awareness for sustainable development and lifestyles in harmony with nature.	12.8.1 Extent to which (i) global citizenship education and (ii) education for sustainable development (including climate change education) are mainstreamed in (a) national education policies; (b) curricula; (c) teacher education; and (d) student assessment.	Consumption (awareness, lifestyles)	attitudes, intentions, behavior, consumers, perceptions, choices, health, nutrition	48
12.a Support developing countries to strengthen their scientific and technological capacity to move towards more sustainable patterns of consumption and production.	12.a.1 Amount of support to developing countries on research and development for sustainable consumption and production and environmentally sound technologies.	Production (technology, capacity building)	impact, future, green	Not addressed
12.b Develop and implement tools to monitor sustainable development impacts for sustainable tourism that creates jobs and promotes local culture and products.	12.b.1 Number of sustainable tourism strategies or policies and implemented action plans with agreed monitoring and evaluation tools.	Consumption (tourism behavior) + Production (tourism industry)	consumers, choices, products, green	Not addressed
12.c Rationalize inefficient fossil-fuel subsidies that encourage wasteful consumption by removing market distortions, in accordance with national circumstances, including by restructuring taxation and phasing out those harmful subsidies, where they exist, to reflect their environmental impacts, taking fully into account the specific needs and conditions of developing countries and minimizing the possible adverse impacts on their development in a manner that protects the poor and the affected communities.	12.c.1 Amount of fossil-fuel subsidies per unit of GDP (production and consumption) and as a proportion of total national expenditure on fossil fuels.	Consumption (energy use) + Production (energy sector subsidies) = Both, but primarily Production-side policy	climate-change, policy, impact	Not addressed

* United Nations. (2017). Global indicator framework for the Sustainable Development Goals and targets of the 2030 Agenda for Sustainable Development (Annex to A/RES/71/313).
